# Extreme Environment Sensing Using Femtosecond Laser-Inscribed Fiber Bragg Gratings

**DOI:** 10.3390/s17122909

**Published:** 2017-12-14

**Authors:** Stephen J. Mihailov, Dan Grobnic, Cyril Hnatovsky, Robert B. Walker, Ping Lu, David Coulas, Huimin Ding

**Affiliations:** National Research Council Canada, 100 Sussex Drive, Ottawa, ON K1A 0R6, Canada; Dan.Grobnic@nrc-cnrc.gc.ca (D.G.); Cyril.Hnatovsky@nrc-cnrc.gc.ca (C.H.); Robert.Walker6@canada.ca (R.B.W.); Ping.Lu@nrc-cnrc.gc.ca (P.L.); David.Coulas@nrc-cnrc.gc.ca (D.C.); Huimin.Ding@nrc-cnrc.gc.ca (H.D.)

**Keywords:** fiber Bragg gratings, femtosecond laser, fiber optic sensor, harsh environment sensing, advanced combustor monitoring

## Abstract

The femtosecond laser-induced fiber Bragg grating is an effective sensor technology that can be deployed in harsh environments. Depending on the optical fiber chosen and the inscription parameters that are used, devices suitable for high temperature, pressure, ionizing radiation and strain sensor applications are possible. Such devices are appropriate for aerospace or energy production applications where there is a need for components, instrumentation and controls that can function in harsh environments. This paper will present a review of some of the more recent developments in this field.

## 1. Introduction

Over the past three decades, fiber Bragg gratings (FBGs) have been used effectively as sensors for a wide variety of applications [1]. The advantages that FBG-based optical fiber sensors possess over more traditional electrical sensing methods include their small size, passive nature, immunity to electromagnetic interference and resistance to harsh environments and corrosion. Furthermore, the multiplexing attributes of FBGs that were developed for optical network applications can be exploited in order to create sensor geometries that are quasi-distributed. The FBG sensor typically acts as a temperature or strain sensor depending on the packaging and waveguide architecture that is chosen for a particular application. It can be used to perform simultaneous measurement of strain and temperature and can be multiplexed into a sensor array that can then be used for structural health monitoring of civil structures, for haptic sensing for robot appendages and ‘smart skins’ for ships or aerospace vehicles. Transducers can be used to convert measurands like pressure, corrosion, moisture, vibration and other environmental effects into detectable strain and temperature changes in the optical fiber. 

The FBG device is an optical filter that is created within the core of an optical fiber waveguide using a high-power laser. It reflects a specific Bragg resonant wavelength of light, *λ_B_*, that depends on the grating periodicity, *Λ_G_*, of the laser-induced modulation of the refractive index, ∆*n*, that makes up the grating structure in the waveguide core region. The FBG performs as a band-rejection filter passing all optical wavelengths of light that are not in resonance with it and reflecting those that satisfy the Bragg condition of the core ∆*n*, namely:

(1) $$\lambda_{B}=2n_{eff}\Lambda_{G}$$

where *n_eff_* is the effective index of refraction seen by the guided mode of probe light propagating through the fiber grating. The sensitivity of both the *n_eff_* and *Λ_G_* to the environment surrounding the optical fiber is the basis upon which the FBG can be used as a sensor. External mechanical effects such as fiber compression or fiber elongation directly change *Λ_G_* while the strain-optic effect changes the *n_eff_*. Both of these changes will vary *λ_B_*, allowing the grating to then act as a strain gauge. Similarly temperature induced changes in the glass refractive index through the thermo-optic effect, and the thermal expansion coefficient of the fiber, allow the FBG to act as a temperature sensor. Shifts to *λ_B_* by an amount ∆*λ_B_* in response to a temperature change ∆*T* and a strain ε is given by [1]:

(2) $$\frac{\Delta \lambda_{B}}{\lambda_{B}}=P_{e}\varepsilon +\left[P_{e}\left(\alpha_{s}-\alpha_{f}\right)+\zeta \right]\Delta$$

where *P_e_* is the strain-optic coefficient, *α_s_* and *α_f_* are the thermal expansion coefficients of any material bonded to the fiber and of the fiber itself, respectively, and ζ is the thermo-optic coefficient. In order to make a quasi-distributed sensor, FBGs with different *Λ_G_*’s can be written at different locations along the same strand of optical fiber. Each FBG will then have a different *λ_B_* and can therefore be multiplexed into a distributed sensor web where different stresses or temperatures can be measured at different locations along the optical fiber length. At telecommunication wavelengths, the *λ_B_* will shift with temperature at a rate of ~10 pm/°C in the absence of strain. At a constant temperature, *λ_B_* will shift ~1 nm under an applied strain of 1000 microstrains (µε). FBG interrogators presently available in the market place are used to monitor spectral shifts in the *λ_B_* due to temperature and strain. These interrogators typically have spectral resolutions in the telecom C-band on the order of 1 picometer (1 pm) which would correlate to a minimum detectable strain of ~1 µε/√Hz rms at constant temperature and minimum temperature variations of 0.1 °C in the absence of strain [1].

Traditional FBG sensors are typically inscribed into the Ge-doped cores of silica-based optical fibers that are photosensitive to high-power ultraviolet (UV) lasers [2]. The index change that produces the ∆*n* is the result of a process where a single UV photon is absorbed by the glass and excites oxygen deficiency defect centers (ODC). These defect centers have absorption bands at ~244 nm [3]. It is also likely that densification of the glass matrix occurs alongside the defect formation process during the laser exposure and contributes to the total index change, especially when large index changes (>5 × 10^−4^) are created [4]. These changes in the refractive index are positive and are often referred to as Type I index change. The durability of the Type I index change is temperature dependent. It follows a power law decay rate that is thought to originate from the thermal depopulation of trapped excited states that are created during the grating formation [5]. At high temperatures, thermal absorption by carriers in the shallowest traps allows them to escape and return to the ground state resulting in a reduced index change. The remaining carriers are thus associated with more stable states which can also relax to the ground state if even higher temperatures are applied. As the most unstable carriers decay at lower temperatures, Type I gratings are typically annealed at temperatures higher than their designed operating temperature in order to remove the unstable carriers and obtain long-term stability in their reflectivity.

For applications below 300 °C, UV laser-induced Type I Bragg grating sensors are excellent for use in civil structures, aircraft, naval ships, oil pipelines etc. as ‘smart skin’ sensor webs to measure ‘in situ’ temperature and stress of these structures. At more extreme temperatures (>450 °C) the refractive index change associated with Type I gratings is erased making such gratings unsuitable as sensing elements above these temperatures. 

The writing of FBGs using a high-powered femtosecond pulse duration (fs) source, such as an 800 nm infrared regeneratively amplified Ti:sapphire laser, has several advantages over the traditional ns pulse duration or CW UV-laser approach. Firstly, the fs grating inscription method based on the phase mask approach is similar to those used industrially for UV FBG manufacturing; however the femtosecond method can be applied to any optical waveguide and is not limited to those that are only UV photosensitive. When infrared femtosecond sources (fs-IR) are used, the laser can modify any material that is transparent to low-signal fs-IR radiation [6]. This capability is especially important for FBG inscription through protective polymer coatings of optical fiber which often absorb visible and ultraviolet light. By frequency doubling or tripling the 800 nm output of the high power femtosecond source, FBG inscription was demonstrated in non-photosensitive fibers by also using femtosecond ultraviolet (fs-UV) and visible femtosecond pulses (fs-vis) at 267 and 400 nm respectively [7,8]. FBG sensors can then be fabricated in other optical materials that are more resistant than Ge-doped silica to certain harsh environments. Secondly, by using certain exposure conditions and specific laser wavelengths, FBGs can be written that are thermally stable up to the glass transition temperature *t_G_* of the material. These thermally stable gratings are ideal for high temperature sensing applications. 

There have been a number of previous review papers on the subject of femtosecond laser inscription of FBGs [9,10,11] for sensors [12] including our own review of femtosecond FBGs for harsh environments [13]. In this paper, we update our previous review of femtosecond laser based FBG harsh environment sensors by giving an overview of inscription techniques that are used along with a discussion of femtosecond laser-material interaction, induced index change mechanisms that apply to these inscription techniques and advances in high temperature applications of fs-IR FBGs. Unique qualities of the femtosecond laser induced FBGs that are made by the different methods will be presented along with how they can be applied to different sensing applications for harsh environments. Regenerated Bragg gratings are another kind of thermally stable FBG structure that can be used as an alternative to high temperature stable femtosecond gratings. Their discussion here is beyond the scope of this review. Interested readers are referred to the review articles [12,13] for more information.

## 2. Femtosecond Laser Induced FBGs 

The use of a high-power femtosecond laser source for FBG creation was first demonstrated by our group in 2003 [14]. In that work, the induced ∆*n* of the gratings resulting from the fs-IR laser exposure were shown to be stable at much higher temperatures than conventional UV-laser induced FBGs. The high intensity nonlinear multiphoton processes that occur during the femtosecond laser induced index change are significantly different from the low intensity single UV-photon absorption processes associated with the ns or CW UV-laser exposures used in the first generation FBG technology. These differences give the fs FBG writing process certain advantages over conventional UV-laser FBG inscription, namely the removal of the necessity to use silica-based UV-photosensitive fibers. FBGs can be written into any waveguide material that is transparent to low signal IR radiation, such as: pure silica core (PSC), fluoride (ZBLAN), borosilicate, phosphate, bismuth oxide, chalcogenide, and alumino-silicate glass fibers as well as crystalline waveguides made of sapphire, YAG and LiNbO_3_ [6]. When fs-IR or fs-vis sources are used, the nonlinear nature of the index change process allows for the fabrication of FBGs that are stable up to the glass transition temperature of the host waveguide material. Such gratings are ideally suited for high-temperature and/or harsh-environment applications. 

### 2.1. Mechanisms for Femtosecond Laser Induced Index Change 

The extremely high peak intensities that can be generated by focused fs-IR laser beams can create localized index changes in bulk glasses that have been used to fabricate embedded waveguides [15]. Peak laser beam intensities >10^13^ W/cm^2^ are sufficient to initiate nonlinear light absorption in glass during the laser pulse. This absorption results in a highly localized deposition of energy into an electron plasma that is formed within the focal volume. The energy of the laser-excited electron plasma is then transferred to the lattice of the bulk material after several picoseconds, which then leads to material modification that is permanent. This modification can be in the form of material compaction, the creation of defects, localized melting, nanograting or microvoid formation. Which type of modification that is created depends on the intensity of the laser pulse and laser irradiation conditions (see below).

#### 2.1.1. Formation of the Free Electron Plasma 

**Figure 1:**
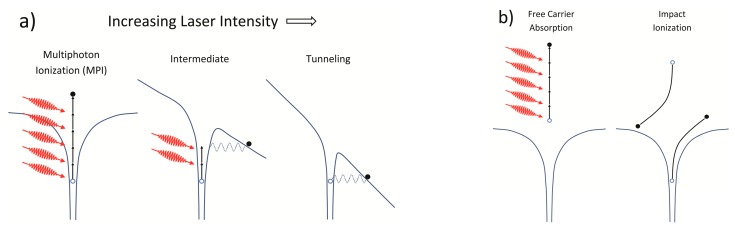
Schematic of free electron plasma formation with high intensity pulses where (**a**) multiphoton and tunneling ionization generates free electrons that (**b**) absorb radiation and impact-ionize surrounding material resulting in avalanche ionization.

Transparent dielectrics do not absorb low intensity incident light, i.e., individual photon energies are insufficient to traverse the material bandgap and be absorbed by the dielectric in a linear way. Within a radiation field of sufficient intensity, many photons can be absorbed simultaneously by a valence band electron resulting in its transition to the conduction band if the total absorbed photon energy is greater than the material bandgap (see Figure 1a). For higher intensities, distortion of the potential barrier between valence and conduction bands as well as the band structure of the material itself can occur leading to nonlinear ionization through tunneling. The relative contribution of these processes to the generation of free electrons depends on the laser intensity and wavelength [16,17]. Electrons now in the conduction band can further absorb incident laser light through free carrier absorption (see Figure 1b). Bound electrons in the surrounding material are then impact-ionized by the accelerated free electrons creating even more free electrons that undergo free carrier absorption and impact-ionize even more bound electrons. This repetitive process, which linearly depends on the laser intensity, continues while the laser field is sufficiently intense, resulting in the generation of an electron avalanche. 

For avalanche ionization to occur, a sufficient quantity of seed electrons is needed in the conduction band. For relatively long laser pulse durations (>10 ps), these seed electrons mostly arises from random impurity or defect states that are thermally excited. This random process results in a non-deterministic fluence threshold (energy/unit area) for modification of the material that is dependent on the square root of the laser pulse duration [16]. For laser pulses shorter than a picosecond, seed electrons for avalanche ionization are generated through the direct nonlinear ionization processes presented above rather than from random thermal excitation. This results in a deterministic threshold fluence for modification of the material. Electrons in the conduction band are heated much more rapidly than energy can be transferred to the lattice. The density of free electrons continues to grow through the avalanche ionization process until the plasma frequency of the conduction band electrons coincides with the incident laser light frequency, after which the plasma strongly absorbs the incident light [17]. The point where the electron plasma becomes strongly absorbing is referred to as the critical plasma density for free electrons, which is on the order of 10^21^ cm^−3^ for near infrared laser light. The incident laser light will continue to be strongly absorbed by the plasma for the duration of the laser pulse. A significant portion of the laser energy is reflected at higher plasma densities. Optical breakdown is assumed to occur when the critical plasma density is reached which for glasses, occurs with intensities on the order of 10^14^ W/cm^2^.

For laser pulse durations shorter than a picosecond, absorbed laser energy does not have enough time to be transferred to the lattice. Significant transfer only occurs long after the cessation of the laser pulse. Energy deposition instead occurs in a shock like fashion, on a more rapid time scale than the material thermal diffusion time. This results in ablation of the material if the laser beam is focused onto the surface or permanent structural change if laser light is focused into the bulk. This is not the case for longer pulses (>nanosecond) where transfer of the energy to the lattice occurs while the laser continues to feed energy into the plasma. The lattice is then catastrophically disrupted well beyond the high-intensity portions of the laser field and classic laser damage ensues.

#### 2.1.2. Induced Index Change Regimes 

It is accepted that femtosecond laser modification of transparent dielectrics includes a stage of nonlinear light absorption followed by free electron plasma formation. However, once electron energy transfer to the lattice has occurred, the subsequent material modification mechanisms are not fully understood. Three main types of laser-induced material modification have been identified: (1) a smooth change in refractive index that behaves in a similar way to the UV-laser induced Type I index change in Ge-doped fibers [18], (2) a form birefringent modification [19] due to self-organized nanostructures [20,21] and (3) material disruption/voids created by light-induced micro-explosions inside the material [22]. Several laser-writing parameters have to be adjusted in order to produce one of the above types of modification. The most important parameters include pulse duration, pulse energy, pulse polarization, pulse repetition rate, the number of pulses deposited in one spot, laser beam focusing conditions (i.e., tight vs. weak focusing) and laser wavelength [23,24,25,26,27]. The peak pulse intensity, which depends on the pulse duration, pulse energy and laser focal spot area, cannot generally be used as a single parameter to unambiguously predict the outcome of the laser-writing procedure.

##### Smooth or Type I Refractive Index Change 

Smooth refractive index change that is induced in bulk silica by 800 nm femtosecond pulses is completely erasable by annealing at temperatures exceeding 900 °C [18]. Micro-Raman spectroscopy studies of silica exposed to high intensity fs-IR radiation have shown that this smooth index change is associated with material densification resulting from an increased concentration of 3 and 4 member rings in the silica structure [28]. The presence of self-trapped exciton defects is also associated with smooth index change as well as a blue emission at 475 nm during laser exposure at inscription intensities of ~5 × 10^13^ W/cm^2^ [29]. The creation of oxygen-deficient (GeE’, SiE’) and non-bridging oxygen hole center (NBOHC) defects was reported during fs IR laser irradiation of bulk Ge-doped silica glass [15]. The same kind of defect formation processes is also important for standard UV-laser inscribed FBGs. 

**Figure 2:**
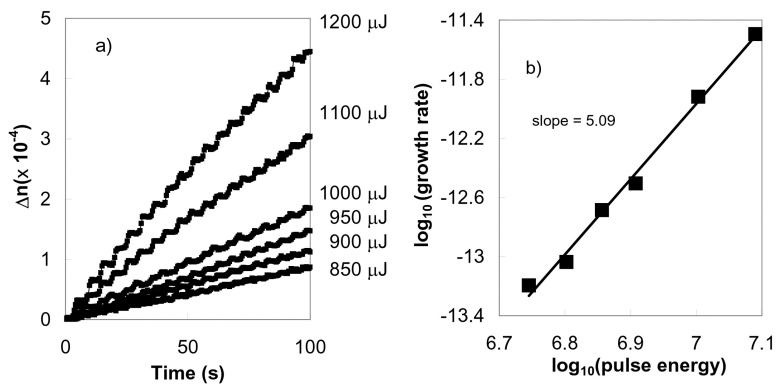
(**a**) Growth of the index modulation ∆*n* as a function of time and pulse energy. (**b**) Scaling behavior of the ∆*n* growth rate as function of energy [30].

FBG inscription in Ge-doped telecommunication optical fibers with a phase mask and multiple pulses of 800 nm fs-IR laser radiation can generate smooth or Type I refractive index change in the fibers. The magnitude of the index change varies with the fifth power of the laser intensity that was used to write the gratings, indicating a 5-photon absorption process [30]. This fifth power scaling dependency between the laser intensity and induced index change is shown in Figure 2. The bandgap for Ge-doped silica having doping levels similar to telecom fibers is ~7.1 eV [31]. This bandgap is much larger than the 1.55 eV energy of a single 800 nm photon. On the other hand, simultaneous absorption of five 800 nm photons would amount to a total energy 7.75 eV which is sufficient energy to bridge the 7.1 eV Ge-doped silica bandgap. Type I-IR and Type I-UV gratings have similar thermal annealing behaviors which suggest that similar defect and/or compaction related mechanisms of induced index change may be occurring in the Type I-IR FBGs in Ge-doped silica.

Densification and color center formation likely contribute to the smooth index change associated with femtosecond laser exposure; however the relative contributions of each process to the index change will vary depending on the glass composition. Hydrogen loading of standard telecom Ge-doped optical fiber, for example, reduces the threshold intensity (under otherwise identical writing conditions) for FBG formation by a factor of 3 in the Type I regime [32]. This result implies that photosensitivity enhancement methods used for UV-laser based FBG inscription in Ge-doped fibers, such as H_2_-loading, high Ge-content cores, etc., can also be exploited by high intensity fs-IR laser systems.

##### Birefringent Refractive Index Change (Type II)

Laser-material interactions that cause dielectric breakdown result in index changes that are permanent at temperatures approaching the material glass transition temperature. For pulse durations >10 picoseconds, dielectric breakdown is also associated with significant material disruption and laser damage. For pulses <1 picosecond, the laser-induced dielectric breakdown can result in refractive index changes that are highly birefringent [19]. This birefringence, which is in fact a form birefringence, is the result of the creation of self-organized periodic nanogratings [20,33]. The orientation of the nanogratings has been shown to be perpendicular to the polarization of the inscription laser and is the result of the absorption of multiple pulses [34]. Such nanograting structure formation has been reported using infrared fs pulses as well as visible fs pulses [35] but has yet to be reported using fs-UV sources. The fabrication of thermally stable gratings based on fs-UV FBGs has not been demonstrated. The fs-UV FBGs have only been reported in the type I regime [36]. 

##### Void Formation

Single tightly focused femtosecond pulses with peak intensities exceeding 10^14^ W/cm^2^ are known to produce microvoids within the bulk glass as a result of confined micro-explosions [22]. Generated pressures in the focal volume exceed the Young’s modulus of the material creating a shockwave after the electrons have transferred their energy to the ions. Timescales for these processes are on the order of 10 ps [37]. Considering conservation of mass, the shockwave leaves behind rarefied material or a microvoid which is surrounded by higher density material having a higher refractive index. 

### 2.2. Femtosecond Laser-Based FBG Inscription Techniques 

#### 2.2.1. Bulk Interferometers

**Figure 3:**
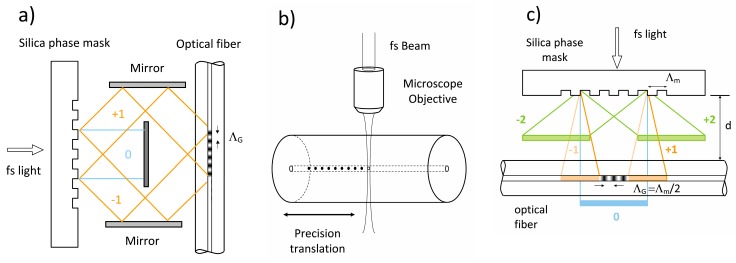
Schematics of principle techniques used for fs-laser inscription of FBGs: (**a**) Talbot interferometer, (**b**) point by point and (**c**) phase mask. Arrows denote period *Λ_G_* of the resultant grating. Amber in the figures denotes propagation of the ±1 orders, green ± 2 order and blue the zero order.

As in the case for the development of UV laser-based inscription of FBG [2], three main techniques for Bragg grating inscription using fs-IR laser sources have been reported. The first demonstrated approach used a bulk interferometer to create periodic index changes in bulk silica [38]. This approach was never applied to grating fabrication in waveguides but it has the advantage of being able to fabricate different grating periods depending on the incident angles of the overlapped femtosecond beams. However, the path lengths of the two interfering beams need to be matched to within the spatial envelopes of the propagating femtosecond pulses in order to insure pulse overlap and creation of an interference pattern. For example, the spatial envelop of the electro-magnetic field of a 30 fs pulse is only 10 µm. The path lengths of the two interfering beams would therefore need to be matched to within 10 µm. In practice, this is very difficult to achieve within a bulk interferometer especially within a manufacturing environment. The Talbot interferometer (See Figure 3a) is a variation of the bulk interferometric approach where a phase mask acts as the beam splitter [39] relaxing some of these alignment tolerances. It has been used successfully to inscribe FBG with femtosecond UV and visible sources [40]. 

#### 2.2.2. Point-by-Point Grating Inscription

The second method for femtosecond laser inscription of FBGs uses a ‘point-by-point’ (PbP) writing technique where individual fs-IR laser pulses are focused into the fiber core using a high numerical aperture (high-NA) microscope objective (see Figure 3b). Highly localized changes to the refractive index are created with each pulse resulting in void formation at the irradiated spot. These localized voids or points are seen by the propagating mode within the optical fiber as a FBG ‘plane’. By translating the beam or optical fiber using high-resolution mechanical translation stages, grating points are created sequentially in a step and repeat fashion [41]. Martinez et al. [41] achieved a 300 nm spot size at 800 nm with which to write a second order Bragg grating in SMF fiber having a 1.075 μm pitch. The ensuing structure is thermally stable, similar to Type II damage UV gratings [42] and to gratings written in the type II regime with the fs-IR laser and the phase masks [30]. By scanning the focal spot across the core, the inscription of distinct grating planes as opposed to points was demonstrated [43,44].

#### 2.2.3. Phase Mask

From a manufacturing perspective, the phase mask approach developed for UV laser FBG inscription is ideal since path lengths of the interfering beams (±1 orders) are matched automatically, significantly reducing alignment tolerances as compared to the bulk interferometric approach discussed previously. When laser sources with low spatial coherence such as excimers are considered, this reduced alignment tolerance is especially important for creation of the interference pattern used to write the grating [45]. When using conventional excimer laser sources for example, path lengths need to be matched to within the ~100 µm spatial coherence length of the excimer source. Similar path length matching (30 µm for a 100 fs pulse) is needed when a femtosecond pulse duration laser source is considered. 

The first demonstration of FBG fabrication using a femtosecond laser source was reported by our group in 2003 using a specialty phase mask. It was similar in design to standard silica phase masks used for UV-laser inscription however it was precisely etched to maximize coupling of the incident 800 nm IR laser radiation into the ±1 orders [14]. With the fs-IR beam aligned at normal incidence to the mask, the path lengths of the generated phase mask orders were automatically matched to within the dimensions of the spatial envelopes of the femtosecond pulses. The resulting sinusoidal interference field created a modulated FBG structure in the fiber that was non-sinusoidal due to the processes of nonlinear induced index change. Higher order filter responses beyond the fundamental Bragg resonance are produced by such non-sinusoidal modulated gratings. An expression for higher order Bragg resonances is given in Equation (3):

(3) $$m\lambda_{B}=2n_{eff}\Lambda_{G}$$

where *m* is the harmonic order number [46]. Creation of Bragg gratings that have higher order resonances at wavelengths of interest can have certain advantages. A phase mask that produces a higher order resonance, say in the telecom C-band at 1550 nm, will have a grating pitch *Λ_G_* that is an integer multiple times larger than the fundamental Bragg resonance pitch *Λ_G_* of 535 nm. The size of *Λ_G_* of the fundamental resonance Bragg grating approaches the resolution limit of most optical microscopes. A higher order grating pitch is easily visualized under an optical microscope. The disadvantage of using higher order resonance gratings is that the index modulation profile is dominated by the main Fourier component associated with the high order structure [47]. To create a certain high order grating reflectivity in the telecom band for example, a much stronger Bragg resonance appears at the fundamental resonance of the structure in the mid-IR. 

##### Phase Mask Longitudinal and Transversal Order Walk-Off

**Figure 4:**
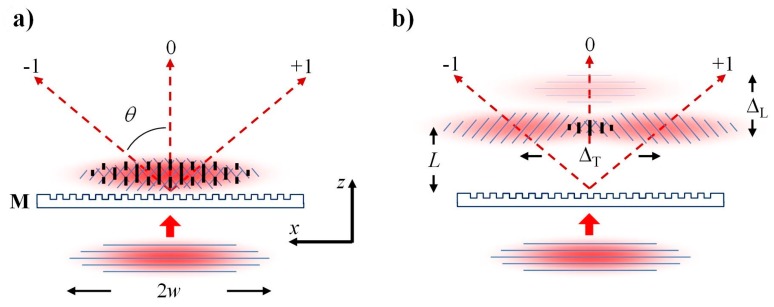
Interference of ultrashort pulses after a phase mask that produces only the 0th and 1st diffraction orders. (**a**) A complex Talbot-like interference pattern formed near the phase mask (M) when the distance *L* from M is small. (**b**) Transverse (∆_T_) and longitudinal (∆_L_) walk-offs become pronounced as *L* is increased. The pulse phase fronts are schematically depicted with blue lines.

Compared to ns pulses or continuous wave (CW) UV-beams, femtosecond pulses interact differently with phase masks. When an incident femtosecond pulse beam is diffracted by the mask, the resultant ultrafast diffracted beams propagate away from the mask surface at different angles. The propagation distance of the diffracted pulse from the surface is dependent on the diffraction order and therefore appears differently when it is projected onto the normal to the mask, as is shown in Figure 4. The femtosecond pulse envelopes of the diffracted beams have different arrival times at a given distance *L* normal to the mask. If *L* sufficiently large, the diffracted order pairs (0, ±1, ±2, etc.) no longer overlap, resulting in a longitudinal walk-off of the diffracted order effect. Consider, for example, a *Λ_m_* = 3.213 μm and a 125 fs pulse, where *Λ_m_* is the pitch of the mask. The ±1 orders generated by the mask diffract away at an angle *θ* from the normal of 14.4°. The spatial separation of the ±1 orders from the zero order and ±2 orders would occur at fiber-mask distances *L* > 1.3 mm for a 100 fs pulse. Small *L* values (i.e., near the mask) result in the overlapping of many or all of the diffracted orders. This overlapping generates a complex interference field pattern with a periodicity that is the same pitch as the mask *Λ_m_*. This periodic pattern undergoes a π-phase shift at a specific distance from the mask [48] that is known as the Talbot length which is defined to be *Z_T_* = 2*a*^2^/*λ* where *a* is the mask pitch and *λ* is the illuminating wavelength [49]. The resulting FBG then has a periodicity *Λ_G_ = Λ_m_* within the Talbot length from the mask. For large values of *L*, only a single diffracted order pair interferes, for example ±1 order pair, because of the longitudinal diffracted order walk-off effect. This results in a pure two-beam interference fringe pattern [50]. The induced index change process in a fiber is highly nonlinear, thus the intensity of the 0 order is too low to induce an index change in the fiber on its own and does not contribute to the final grating pattern in the waveguide. Usage of the longitudinal walk-off effect has the clear advantage that for femtosecond pulses, the phase mask need not be zero-order suppressed in order to produce a pure 2-beam interference pattern that can be imprinted on the fiber. Employment of the longitudinal order walk-off effect results in the interference of the ±1 orders alone which produces a grating period *Λ_G_ = Λ_m_*/2.

It is important to note that while the longitudinal walk results in the isolation of a diffracted order pair from other orders, transverse walk-off results in a reduction of overlap of the ordered pair. For example, a phase mask designed to produce a grating with a fundamental Bragg resonance in the telecommunication band of 1550 nm in a standard telecom fiber will have a pitch of ~1.07 µm, which will produce a FBG pitch in the fiber of 535 nm if only the ±1 orders interfere. If using an 800 nm femtosecond source, the diffraction angle *θ* of the ±1orders diffracted by the phase mask will be quite large (~49°), which can result in significant reduction in diffracted beam overlap, especially if the diameter of the incident beam on the mask is small. 

The PbP technique, by its nature, is more versatile than the phase mask approach for creating complex structures like multiple π-phase shifts, sampled gratings, anti-symmetric gratings, etc. [51] as grating structures are easily changed on the fly by varying laser pulse repetition rate, stage velocity and beam focusing position. The resulting structures are necessarily thermally stable as a result of the microvoid formation. The disadvantage of the PbP technique when compared to the phase mask approach is the reproducibility of a given structure from a manufacturing perspective. The phase mask approach allows one to manufacture similar complex grating structures that are identical and thermally stable, however a dedicated phase mask is required for each structure. It is easier to inscribe gratings in the Type I regime with the phase mask approach, which is important for creating gratings with low scattering loss and high mechanical reliability especially when written directly through the protective polymer coatings of fibers. Such through-the-coating (TTC) gratings are important for measuring high mechanical strains.

#### 2.2.4. Phase Mask Bragg Resonance Tuning for Distributed FBG Array Fabrication

A clear disadvantage of the phase mask approach to FBG inscription when compared to the PbP approach is that the grating pattern created in the fiber is defined by the mask pitch *Λ_m_*. In a typical production environment, an inventory of phase masks is maintained in order to manufacture gratings with different Bragg resonances say for a distributed sensor array. Each phase mask can be very costly. Changing and aligning of masks to produce different grating resonances on a production line can be a time-consuming and tedious process. It is therefore desirable to have a capability to fabricate gratings with different Bragg resonances from a single phase mask, without having to perform a complicated alignment procedure especially for the fabrication of distributed sensor arrays.

Several techniques have been developed to manufacture Bragg gratings with different periods *Λ_G_* from a phase mask having only one fixed period *Λ_m_*. Inscription of a grating with a phase mask while the optical fiber is under tensile strain will result in a *λ_B_* that is shifted to lower wavelengths as compared to a FBG made using the same phase mask in unstrained fiber [52]. Only a small amount of strain can be tolerated by the optical fibers especially if the FBG is inscribed in the Type II regime, so the amount of *λ_B_* adjustment by applying fiber strain is limited. For Bragg gratings written in standard single-mode germanium-doped telecom fiber in the telecom band, the achievable tuning of *λ_B_* is less than 2 nm. 

Another approach for changing the Bragg grating period from a fixed mask involves the placement of a simple lens in the beam path before the phase mask. The lens introduces a curvature on the wavefront of the diffracting light that results in a magnification at a distance of the original periodicity produced by the mask [53]. To produce a magnification effect of any significance however, the phase mask to fiber distance needs to be at least several millimeters. This distance is difficult to achieve with UV laser systems such as excimers because of their short spatial coherence length. This approach was demonstrated however using femtosecond IR beams [54,55] where the creation of a non-collimated femtosecond beam along the fiber axis resulted in wavelength shifts of Bragg gratings in the telecom band of up to 40 nm. Curvature to the wavefront of light diffracted by the phase mask could also be accomplished by using a spatial light modulator to modify the wavefront before the mask. In this way a 35 nm tuning range of the *λ_B_* in telecom band was achieved [56]. Similarly modification of the curvature of the wavefront of light diffracted by the phase mask was achieved by replacing the spatial light modulator with a deformable mirror and a convex lens [57]. In this instance wavelength tuning of the *λ_B_* in telecom band of up to 11 nm was achieved.

The phase mask approach has also been successfully demonstrated using frequency doubled 400 nm femtosecond pulses for inscription of fine pitched FBGs for Yb fiber laser cavity mirrors [8] as well as with frequency tripled Ti:sapphire laser pulses at 267 nm [58] for inscription in pure silica fibers [7]. With the femtosecond pulse wavelength shifted to the UV however, the direct phase mask approach is not appropriate for type II fs-UV inscription. The direct phase mask approach requires refractive optics (phase masks, focusing lens etc.) which contribute significant amounts of dispersion to the fs-UV pulse. For type II formation, high intensities would also be required with the fiber placed in close proximity to the silica phase mask. This combination of high laser intensity coupled with high photon energy may result in phase mask degradation or damage through the rapid generation of absorbing colour centre defects [40]. To date there has been no report of type II fs-UV FBG.

## 3. Applications of fs-Laser Induced FBGs for Sensing

Traditional UV-laser written FBG sensor arrays are used to measure static and dynamic strain for structural health monitoring of aerospace, naval and civil structures. The oil field servicing companies in the oil and gas sector are using distributed FBG sensors for temperature and pressure monitoring of oil well downholes and reservoirs where harsh environments of 20 kpsi and 185 °C are common [59]. However, as new unconventional oil extraction techniques based on thermal recovery methods are being developed, such as steam assisted gravity drainage (SAGD), cyclic steam stimulation (CSS) or toe-to-heel air injection (THAI), traditional UV-laser based FBG sensors are unable to withstand the higher temperatures associated with these methods (up to 600 °C) as the FBG spectra quickly erase and disappear [60]. Fiber optic sensors are also hindered by fiber losses caused by ingress of hydrogen gas that occurs within the downhole environment. Standard Ge-doped telecom fibers that are typically used in fiber optic sensing platforms are especially hampered by this effect. PSC fibers are an attractive alternative for this application in that they do not suffer from hydrogen-induced attenuation and can be used to mitigate these effects [60]. However, production of distributed FBG sensor arrays using conventional UV-laser based inscription is extremely difficult in PSC fiber. High photon energy 193 nm ArF excimer laser radiation can be used to write FBGs in PSC fibers [61] but only after lengthy writing exposure times which degrades fiber reliability. Long writing times are also not practical from a manufacturing perspective. The fs approach on the other hand can easily produce high ∆*n* FBGs in PSC fibers that are resistant to hydrogen induced loss [7,62]. Natural gas turbines, entrained coal gasifier or aerospace turbine monitoring require sensors that can operate at temperatures above 1600 °C [63]. While fs-vis and fs-UV FBGs have been fabricated for some sensing and fiber laser applications, they have not been generally fabricated for sensing in high temperature environments.

### 3.1. High Temperature

High temperature stable FBGs can be fabricated using either the PbP or the phase mask method which results in gratings that are suitable for applications in harsh environments such as those found within power plants, gas or aerosapce turbines, combustion systems, etc. When the PbP method is used for FBG inscription, the intensity threshold for Type II index change is necessarily traversed in order to produce sufficient index change with a single pulse. The resulting microvoid structure results in an index perturbation or grating ‘plane’ that is thermally stable at least up to 1000 °C in standard telecom fibers [42]. 

**Figure 5:**
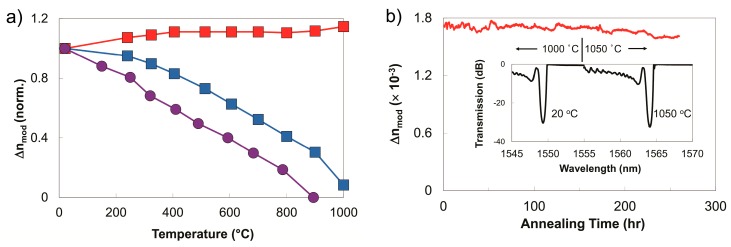
(**a**) Short-term annealing of Type II-IR (red), Type I-IR (blue) and Type I-UV (violet) grating held at each temperature for 1 h; (**b**) grating reflectivity expressed as its index modulation (∆n_mod_) for a thermally stable grating (red) as a function of time. (Inset) Grating spectrum as a function of temperature.

In the case of the phase mask method, thermally stable Type II gratings can be made using a multi-pulse exposure. Nanograting formation, which is associated with thermally stable Type II index change, is more likely when the Fourier transform limited femtosecond pulse is chirped beyond 200 fs and multiple pulses are used [27]. The peak intensity reduction caused by the temporally chirping of the pulse can be mitigated by placing the fiber in close proximity to the phase mask thus preventing the order walk-off effects. The resulting complex interference field can have higher peak intensities than the sinusoidal interference pattern associated with two beam interference [48]. Long term thermal stability and reflectivity of fs-IR FBGs written in this fashion in both Ge-doped telecom fiber and non-UV photosensitive PSC (fluorine-doped silica clad) fibers were studied [64]. Higher order resonance FBGs with large ∆*n*’s were written in both fiber types and in both the Type I and the Type II regimes using a Ti:sapphire regenerative amplifier with 1 mJ pulse energies that were chirped from transform limited 100 fs pulses to 500 fs for the Type II cases. A standard UV laser-induced FBG was fabricated with a frequency doubled argon-ion laser. Isochronal annealing curves up to 1000 °C for the devices are presented in Figure 5a, where device temperatures were allowed to stabilize at each temperature for one hour. Both Type I gratings resulting from either the UV and fs-laser exposures anneal during the process with complete erasure being observed for the case of the UV-laser-induced FBG. For the Type II FBG, an increase in ∆*n* was observed, which corresponded to a modest increase in grating reflectivity. This increase is likely a result of simultaneous induction of both Type I and Type II index changes during the femtosecond laser inscription. The peaks of the complex interference pattern are sufficiently intense to produce a Type II structure in the fiber however some Type I index change is also generated in the valleys of the interference pattern, where the intensity was too low for Type II formation. During the grating annealing process, the permanent Type II index change remains stable while the erasable Type I index change is removed resulting in a higher ∆*n* contrast.

The Type II process creates a gradient in the index change across the fiber core such that higher changes occur at the core/cladding interface of the fiber. This gradient generates a ghost mode and cladding mode coupling losses in the transmission spectrum that are observed at shorter wavelengths than the Bragg resonance (see Figure 5b). The cladding mode losses associated with Type II fs-IR gratings differs from those of Type I fs-IR gratings, where in the latter case, the index change can be induced uniformly across both the Ge-doped core and pure silica cladding of telecom fibers resulting in transmission spectra that do not have out-of-band cladding mode losses [65]. Sustained high temperature performance of the Type II grating is shown in Figure 5b where the temperature was maintained at 1000 °C for 150 h. No significant reduction of the grating reflectivity was observed for the duration of the test, with the grating maintaining a ∆*n* = 1.7 × 10^−3^. The FBG temperature was then raised and kept at 1050 °C for 100 h during which time the ∆*n* fell slightly from 1.7 × 10^−3^ to 1.6 × 10^−3^. Spectra taken initially at room temperature and after 100 h at 1050 °C are shown in inset of Figure 5b. A drift in *λ_B_* of 0.2 nm to longer wavelengths was detected at the end of experiment. Pre-annealing of the fiber at high temperatures relaxes residual stresses that result in higher temperature stability of Type II fs-IR FBGs up to 1200 °C [66] for time durations of several hours. 

**Figure 6:**
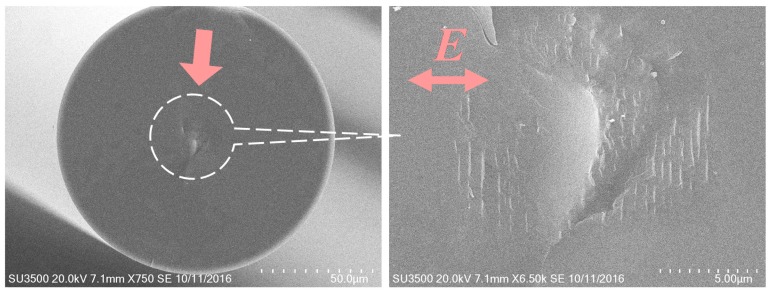
Nanograting nature of Type II-IR FBGs in SMF-28 fiber, as revealed using SEM. The figure on the left shows the cross section of optical fiber with a Type II-IR grating present. The right figure is a magnified view of the modified portion of the fiber in the left figure. The nanogratings were produced using chirped 150 fs pulses, a NA = 0.2 cylindrical lens and a 3.21 μm pitch mask. The pulse energy was 900 μJ.

An important issue with the single pulse PbP gratings is that the induced index change is the result of microvoid formation. It has been recently shown that Type II FBGs written with the phase mask in the multi-pulse irradiation regime are instead comprised of nanograting structures which scatter significant amounts of visible light when interrogated using dark field optical microscopy [67]. To reveal the nanoscale morphology of a Type II FBG written in Ge-doped standard telecom fiber, a Type II FBG is cleaved perpendicular to the fiber axis and coated with 200 Å of gold/palladium and then examined using a scanning electron microscope. Figure 6 displays a scanning electron micrograph of the nanograting structure seen in the fiber core. Similar to microvoids, nanograting structures result in high scattering losses generally which makes it difficult to concatenate gratings within a single fiber in order to make a distributed sensor array. It has recently been observed that while using the phase mask technique and exposure parameters for Type II grating inscription (pulse energy and duration) at low pulse repetition rates (i.e., 1–5 Hz), which makes it much easier to follow the grating evolution, Type II grating formation is preceded by the initial formation and subsequent erasure of a Type I grating structure with sequential writing pulses [68]. It was found that if the inscription process was ceased when Type I grating growth saturated and erasure would start with subsequent pulses, then a post thermal treatment resulted in the formation of a typical Type II grating, with its associated cladding mode generation, but with very low insertion loss. Many such thermally stable gratings could then be concatenated into distributed sensor arrays.

#### 3.1.1. Oxy-Fuel Fluidized Bed Combustor Monitoring 

Advanced power plant technologies such as oxy-fuel fluidized bed combustion, chemical looping combustion, and entrained flow gasification, are in development to reduce greenhouse gas emissions [69]. However, the operation and performance of these technologies are limited by conventional sensors and controls, which are unsuitable for the high temperature, pressure and corrosive conditions present. Similarly, for gas turbine engine development, accurate combustor and exhaust measurements are increasingly important since demands for higher efficiency and more stringent emission targets drive operation closer to established limits. Femtosecond laser written Type II silica based FBG sensor arrays were fabricated using the phase mask approach and deployed in a number of combustor systems used for power generation [70,71] and transportation [72]. 

**Figure 7:**
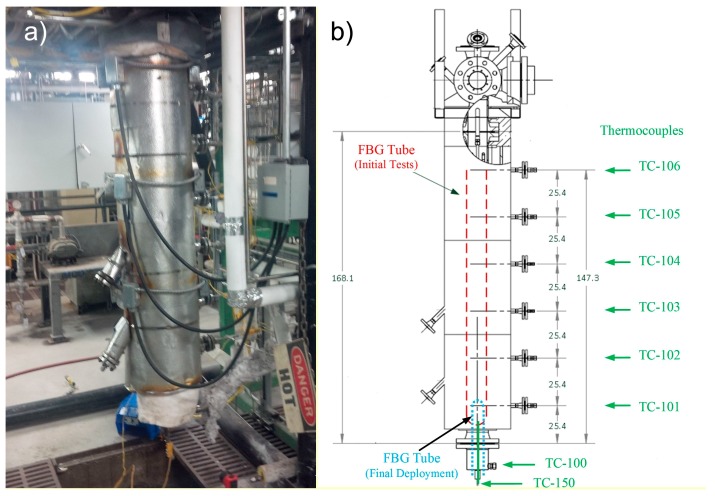
Bottom section of fluidized bed combustor (with insulation). (**a**) Photo, and (**b**) Depiction of internal FBG and thermocouple locations.

For the example of the oxy-fuel fluidized bed combustor deployment [70], a sensor array comprising 14 second order Type II FBGs in standard SMF fiber was fed through a 1/4inch outer diameter (OD) 310 stainless steel tube that was centrally located in the combustor chamber as depicted by the red dashed line of Figure 7b. A single grating written in PSC fiber was also deployed in the same tube. Additionally, seven arrays with a total of 132 fs-IR laser written Type II FBG temperature sensor elements were deployed in a similar tube located as shown by the blue dashed line of Figure 7b. Eight ports are also shown where thermocouples extend just inside the inner reactor wall. Typically, gratings for these tests were annealed in-situ during normal combustor operations. For these tests, a SM125 4-channel FBG interrogator (Micron Optics, Atlanta, GA, USA) was used to log the reflection spectra, track Bragg resonances and display sensor temperatures graphically in real time. 

**Figure 8:**
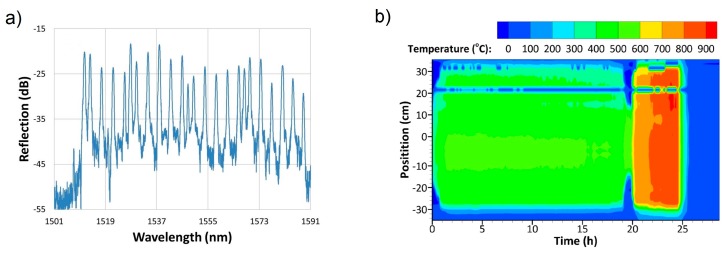
(**a**) Room temperature reflection spectra from a single fiber array after one thermal cycle of combustor; (**b**) Fluidized bed combustor temperature as a function of position and time, during part of one thermal cycle, plotted with 10-min resolution. Data from 3 fiber arrays with 2-cm sensor spacing are shown.

Figure 8a presents a single fiber FBG sensor array reflection spectrum. Figure 8b presents the fluidized bed combustor temperature profile as a function of position and time during a portion of one thermal cycle of the combustor. Multiple thermal cycles up to 1025 °C performed over a five month period. Good agreement is observed with point thermocouple data, but additional detail in thermal profiles are obtained through the use of the distributed FBG sensor array. When compared to the thermocouple approach to distributed temperature measurement, this fiber optic deployment demonstrated clear advantages in terms of sensor cable management, ease of installation, redundancy and thermal response speed.

#### 3.1.2. Gas Turbine Combustor Simulator Monitoring

Gas turbine monitoring using thermally stable FBG sensor arrays facilitates accurate measurement of hot gas working temperatures within a turbine, which is the critical control parameter that is essential for safe, reliable, efficient, and cost-effective operation. Overheating and considerable damage to turbine blades can be caused by inhomogeneous combustion; however accurate measurements of the blades and vanes within a gas turbine are very difficult [73]. Nonetheless, measurement of gas temperatures in the turbine exhaust path or combustor liner can also provide important insights, measures of performance and potential boundary condition inputs for thermal models. To this end, a gas turbine combustor simulator is monitored using FBG grating arrays similar to those used previously for the oxy-fuel fluidized bed combustor. Grating arrays were packaged in two temperature sensing probes, constructed from standard 316 stainless steel tubing. The diameter of one probe was 1/16inch and the other was 1/8inch. Shrink tubing was used to secure two fiber arrays (42 FBGs total) in each tube, staggered roughly 2.5 mm relative to one another. Following packaging and prior to installation, each probe was annealed in a tube furnace, using multiple cycles to 1100 °C.

**Figure 9:**
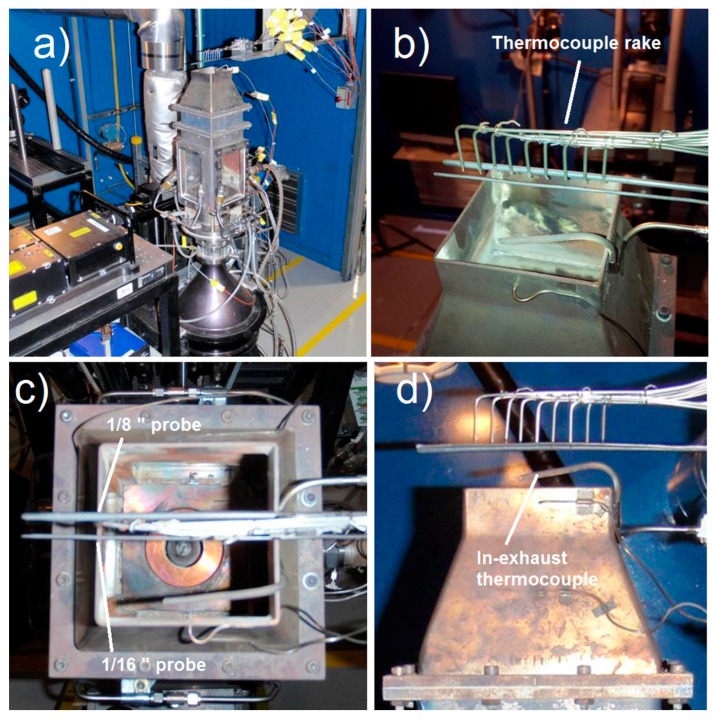
(**a**) Image of the gas turbine combustor simulator with (**b**) angled close up view of the exhaust region; (**c**) top view of the exhaust region and (**d**) side view of the exhaust region with thermocouples and FBG sensor probesdenoted in each figure.

Initially, exhaust temperatures were measured. For this phase of the experiment, the setup was as depicted in Figure 9, showing the gas turbine combustor simulator, along with the FBG sensor probes and rake of open-ended thermocouples. The outer edge of the exhaust nozzle measures 83 mm square, with a thickness of 2 mm. The FBG sensor probes were placed axially across the combustor exhaust, separated laterally by 8.8 mm. Nine open-ended thermocouples were centered in between and distributed in the axial direction with a separation of 9.5 mm. During the experiment, the combustor simulator was translated relative to the sensors, such that the sensor probe positions were varied across the exhaust nozzle from −60 mm to +60 mm. For these tests, a Micron Optics SI255 Hyperion 16-channel FBG interrogator was used to track the Bragg resonances at a frequency of 1 kHz.

**Figure 10:**
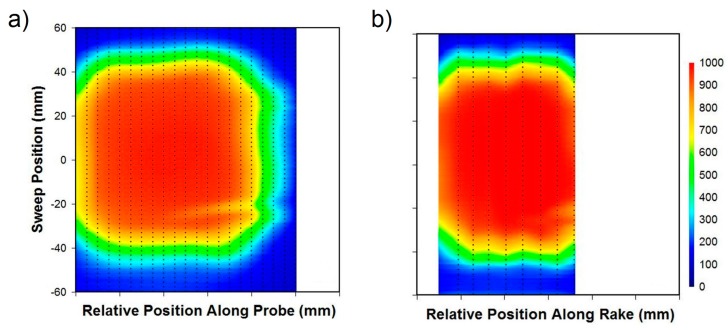
Temperature measurement of the gas turbine combustor simulator exhaust region using (**a**) the FBG sensor array and (**b**) the thermocouple rake. Sensor positions are denoted by the vertical dotted lines.

Figure 10 contrasts the data collected from the 1/16” FBG sensor probe with that of the thermocouple rake, both swept at constant velocity of 0.125 mm/s across the exhaust aperture. The presence of the in-exhaust thermocouple (identified in Figure 9) is clearly visible in the lower right corner of the thermal image in Figure 10a. The result also compares well with that obtained using the thermocouples (Figure 10b). Since a greater density of FBGs is present, it is not surprising that the FBG contours appear smoother and more highly resolved. Although the FBGs under reported temperatures by 5 to 10% in some cases, this can be attributed to calibration and packaging issues. Given that the wavelength to temperature calibration curves were established for unpackaged FBGs [71], the effects of heat transfer due to the package’s thermal conductivity have not been included. These differences can therefore be corrected through re-calibration and by improving the package design to reduce thermal wicking.

**Figure 11:**
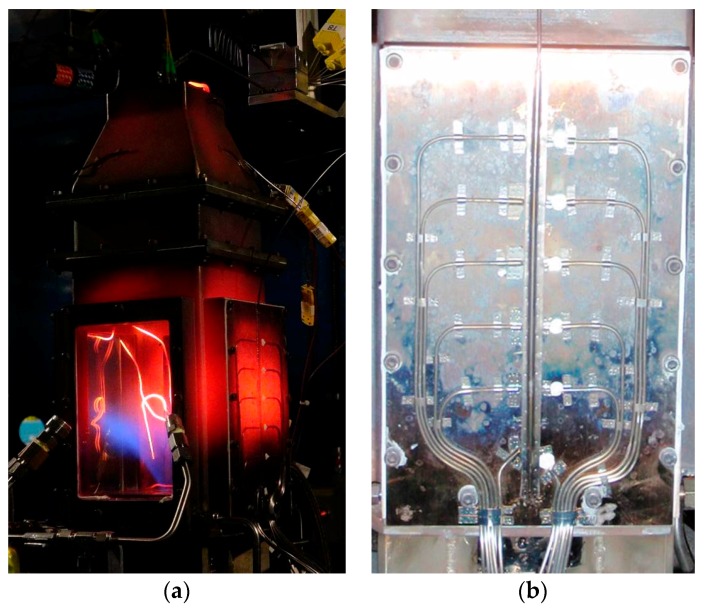
(**a**) Gas turbine combustor simulator operating with sidewall measurement panel, thermocouples and 1/16” tube packaged FBG sensor probe; (**b**) close up view of the sidewall panel at room temperature.

**Figure 12:**
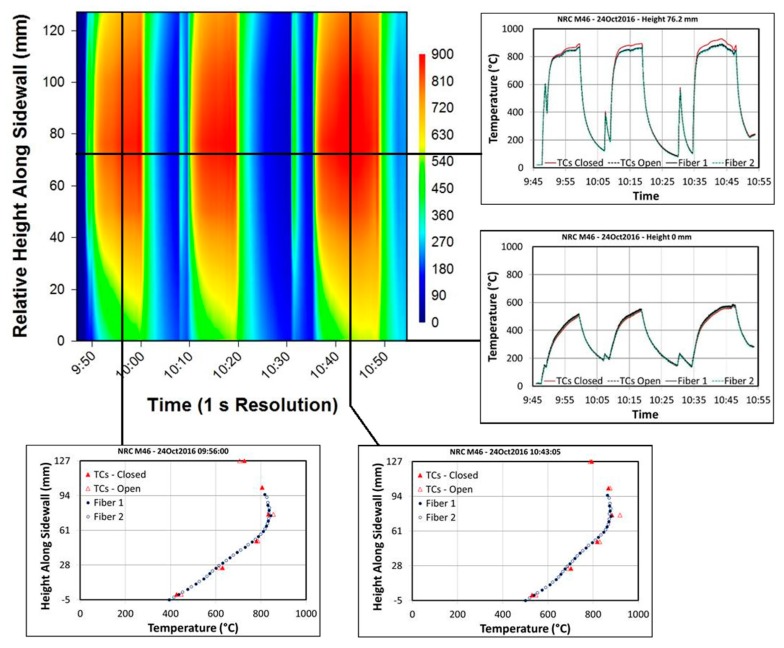
Comparison examples of sidewall temperature measurements at various points in time and space.

Following the exhaust temperature measurements, one of the fused silica windows in the combustor simulator was replaced with the sidewall measurement panel shown in Figure 11. The panel was fitted with six closed-ended thermocouples (on the left) and six open-ended thermocouples (on the right), separated laterally by 3/8” and spaced 1” apart vertically. The open ended thermocouples were bonded to the panel using a ceramic bonding agent, with all remaining probes and wires held in place by nichrome strips, spot welded to the panel. The same 1/16” FBG probe used to measure exhaust temperatures was installed between the two lines of thermocouples. The combustor simulator was cycled through a range of temperatures, with various quantities of cooling air injected at the bottom in order to manipulate the vertical thermal gradient. The sidewall measurements were conducted over approximately one hour. Figure 12 displays a standard distributed temperature sensing (DTS) plot of the sidewall temperature gradient history. The results are very similar between the FBG sensor array and the open ended thermocouple probes. Figure 12 also provides some example curves extracted from the DTS plot for closer analysis.

#### 3.1.3. Type II FBGs in Fiber Canes

An important consideration for any high temperature silica-based fiber optic sensor is that of sensor packaging. Unpackaged standard silica single mode fibers that are kept in air at temperatures near or above 1000 °C loose almost all of their mechanical integrity. While the fibers themselves can survive for hundreds of hours at 1000 °C when left undisturbed, they experience severe mechanical degradation and embrittlement when placed in oxidizing atmospheres at high temperature. Any strain applied to the fiber after such an exposure will cause it to shatter [64]. This embrittlement will also create issues if a fiber is heated to extreme temperatures in air while under strain due to say its packaging architecture. Protection of the fiber from oxygen exposure at high temperature is possible by using a suitable package that itself survives the high temperature. Application of or inscription through a coating on the fiber is the most obvious choice for fiber protection. For higher temperature applications metallic coatings are preferred, with gold coatings rated for the highest temperature operation at ~700 °C. This rating, however, is not suitable for temperatures close to 1000 °C [74]. Stainless steel or ceramic alumina tubing is used as a packaging option however it is less desirable when the fiber sensor needs to be integrated into composite structures or deployed within a confined space.

**Figure 13:**
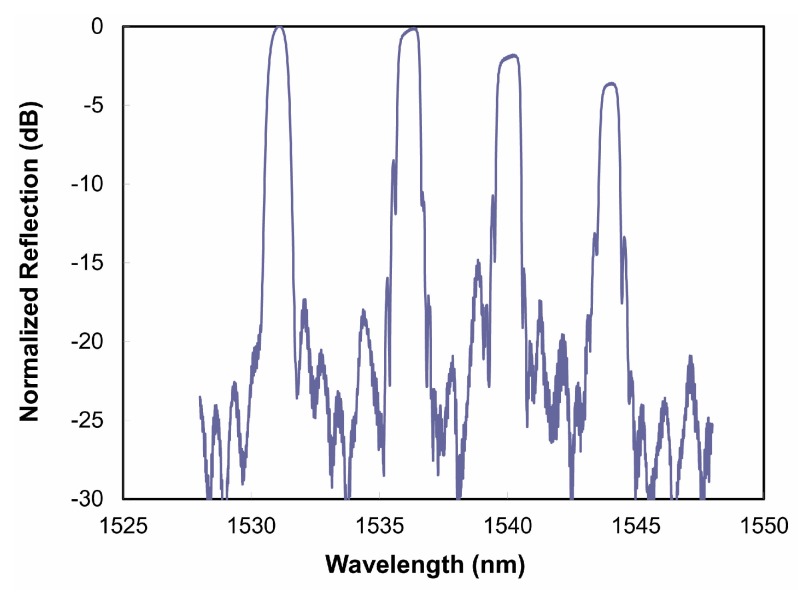
Reflection spectrum of a 4-element Type II FBG sensor array written in 400 µm clad single mode fiber.

A Type II grating array was made in the core of a fiber cane using the fs-IR laser/phase mask approach [75]. The fiber cane was a single mode optical fiber with an 8 μm core diameter but with a 400 μm cladding diameter. The mechanical strength of the fiber cane Bragg grating was evaluated after the long term annealing in a tube furnace in air at 1020 °C for 150 h. The sensor array spectra taken at room temperature after the 150 h of annealing are shown in Figure 13. After the long term annealing, the 400 μm clad fiber maintained enough mechanical integrity to be handled. The FBG sensor array was subsequently proof tested to ~39 MPa maximum stress. The pristine fiber was proof tested by the manufactures to 690 MPa. Because of the large outer diameter of the fiber cane, it is more rigid than a standard 125 μm diameter SMF fiber but it can still accommodate bending radii of less than 10 cm, making it suitable for the many of sensing applications.

#### 3.1.4. Type II FBGs in Microstructured Fibers

Long term annealing of FBGs in standard single mode silica fibers at temperatures between 1100 °C and 1200 °C however is likely to induce dopant migration within the core of standard optical fibers, which will result in a continual change of the fiber *n_eff_* and, as a consequence, a continual shift of the Bragg resonance. The fabrication of femtosecond laser induced FBGs in single material silica optical fibers such as photonic crystal fibers [76] or microstructured fibers like suspended core or ‘grape-fruit’ fibers do not suffer from the core diffusion issue. In fact, recently PbP femtosecond laser induced gratings were inscribed through an ablative process on the surface of a suspended core fiber [77]. The 11 µm suspended core supported a few modes in the telecom C-band producing a larger bandwidth of the resultant Bragg grating response which was relatively stable up to 1300 °C.

#### 3.1.5. Sapphire FBGs

**Figure 14:**
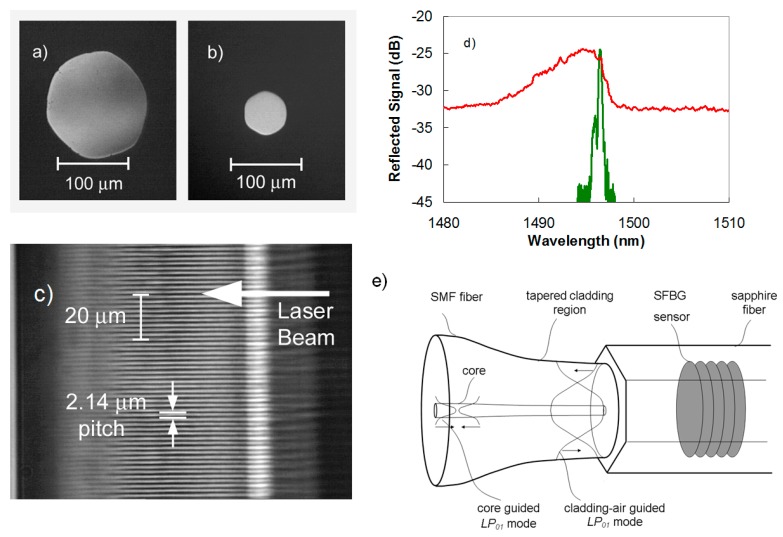
Cross-sections of commercially available (**a**) 125 μm and (**b**) 60 μm diameter sapphire fiber are shown; the grating structure inscribed in 150 μm diameter fibre is shown in (**c**); the corresponding multimode reflection response is shown by the red trace in (**d**); when using the single-mode field expander shown in (**e**), the single-mode reflection spectrum shown in green in (**d**) is obtained.

Silica based optical fibers can no longer function as sensors for temperatures >1300 °C. Single crystal sapphire fiber, which has a glass transition temperature *t_g_* of ~2030 °C, is the most successful optical fiber used for high-temperature sensing applications [78]. Sapphire fibers however are made in the form of a single rod of C-axis sapphire crystal but do not possess a cladding layer, which makes them highly multimode and sensitive to bending losses and mode conversion for fiber diameters that are commercially available. Examples of such fibers are shown in Figure 14a,b. 

Existing sapphire fiber sensors are largely Fabry-Perot structures created within the fiber that produce a broadband interferometric signal that varies with temperature [63]. These devices can be used as point sensors but cannot be used effectively for distributed sensing i.e., measuring at different location along the fiber. 

The phase mask approach has been used successfully for the inscription of high order FBGs in sapphire fiber (SFBGs) [79,80]. It may be possible to inscribe a grating plane using the PbP method as was demonstrated within the cores of silica based fibers [43,44], however it would be very time consuming especially considering that a grating plane would need to subtend the entire cross-section of the multimode sapphire fiber (typically 150 µm diameter) and not just the 8 µm core of a standard single mode fiber. Volume Bragg gratings are more easily inscribed across the majority of the sapphire fiber cross section with the phase mask approach. Recently the PbP method was successfully demonstrated for the inscription of second order FBGs (1.776 μm pitch) in 125 μm diameter sapphire fibers [81]. Although the grating reflectivity was an order of magnitude weaker than what could be achieved using the phase mask approach, the PbP method has the advantage to easily manufacture a distributed FBG array. 

The reflection spectrum of a SFBG is highly multimode with a broad spectral bandwidth several times larger than a similar length uniform pitched FBG in single mode fiber. The broad spectral response is not as sensitive to temperature and strain as its single mode fiber counterpart. A multimode reflection spectrum from a fifth order SFBG sensor when interrogated with a multimode coupler and white light source is presented as a red trace in Figure 14d. The large bandwidth spectral response has a complicated structure that is the superposition of thousands of different modes reflected by the grating. Spectral averaging and numerical techniques have been used to improve the SFBG measurement sensitivity as a function of temperature [82]. The sensitivity of the spectral response to changes in temperature and strain is however much better improved by producing a narrowband single mode response, similar to that of an FBG sensor in standard fiber. Such a single mode response is also more easily processed by conventional FBG interrogators. To produce such a single mode response from a multimode SFBG, the device needs to be probed by exciting the fundamental propagation mode supported by the fiber. This can be accomplished by using an adiabatic fiber taper to expand the ~10 μm diameter single mode into a Gaussian mode with a diameter approaching that of the sapphire fiber (as shown in Figure 14e) [80]. The fundamental guided mode of the sapphire waveguide is then excited producing a single mode reflection response (green trace Figure 14d). With the single mode response, a more accurate measurement of the effective refractive index sapphire fiber was possible. In [80], a phase mask with a 1.747 μm created a second order Bragg resonance in the sapphire fiber at 1525.496 nm. The resulting *n_eff_* for the sapphire fiber is then 1.746.

No reduction in the strength of SFBG is observed at high temperatures up to 1850 °C [83]. By using a fs-VIS pulses (400 nm) and a Talbot interferometer a three element SFBG array was demonstrated where the FBGs were inscribed at the fundamental Bragg resonance [84]. Although not demonstrated in this work, such fundamental Bragg resonance gratings should have higher reflectivities than the higher order gratings reported. The SFBG sensor approach has distinct advantages over other sapphire fiber sensors that rely on Fabry-Perot etalons at the fiber tip. Unlike Fabry-Perot sapphire sensors, SFBG sensors with their discrete resonant wavelength could potentially be used as distributed optical sensor arrays up to 2000 °C.

### 3.2. High Ionizing Radiation Environment

For nuclear applications, electronic gauges and transducers are not suitable for deployment in harsh radioactive environments especially when intense ionizing radiation is accompanied by elevated temperatures, chemical contamination and high levels of electromagnetic interference. Materials can suffer damaging effects as a result of neutron bombardment. For example, when exposed to intense radiation, the two metals forming a thermocouple will transmute into different elements resulting in a drift of the thermocouple output. 

FBG sensors inscribed in Ge-doped optical fiber with UV-lasers can tolerate low flux nuclear environments for extended time durations [85]. However within high flux environments, radiation-induced attenuation (RIA) renders Ge-doped telecom fibers opaque after a period of time. UV-laser inscription of Type I FBGs in Ge-doped fibers relies on the same defect formation mechanisms that are associated with RIA. If one creates a radiation hardened fiber that is resistant to RIA, such as one having a pure silica or fluorine-doped silica core [86], such a fiber is also resistant to Bragg grating formation through the UV exposure process. Traditional UV-laser FBG writing techniques are therefore ineffective. Using fs-IR phase mask approach, both Type I and Type II FBGs were easily written into radiation hardened F-doped fiber [87,88]. In these experiments, only small changes in grating strength and resonant wavelength were observed after a 100 kGy dose of γ-radiation. In other experiments, higher doses up to 1 MGy resulted in a −60 pm shift of the Bragg wavelength [89]. It was also demonstrated fs-IR FBGs that are inscribed into PSC radiation hardened fiber can still function as temperature sensors even after exposure to neutron radiation fluences of 2.83 × 10^19^ n_fast_/cm^2^ [90].

### 3.3. Multiparameter Sensing in Harsh Environments

The primary measurand used in a FBG sensor is *λ_B_* which is affected by changes to the grating periodicity *Λ_G_* either by thermal expansion or strain as well as by changes to *n_eff_* due to changes in the thermo-optic and strain optic coefficients. In a standard optical fiber it is often difficult to discriminate between these two effects with a single FBG since both effects can act simultaneously on *λ_B_*. To differentiate between these effects, another FBG is often placed in close proximity to the first FBG but is isolated from one of the effects. Although effective, it not a true measurement of different parameters at one location and often requires a complicated sensing configuration. Alternatives to the multi-fiber solution to multi-parameter sensing have been proposed mainly for strain and temperature discrimination. They include FBGs superimposed on the same portion of fiber but with different *λ_B_* [91], FBGs in fiber with dissimilar diameters [92], higher order FBG responses resulting from saturated UV-laser exposures [93], FBGs in birefringent fibers [94], etc. Frazão et al. [95] present a comprehensive review of these various methods.

**Figure 15:**
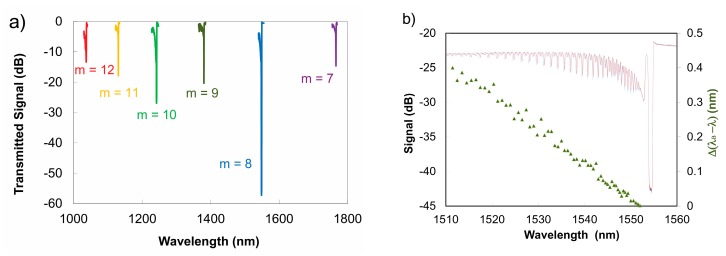
(**a**) Transmission spectrum of a type II-IR grating written in 980 nm cut-off single mode fiber with the 4.28 μm pitched phase mask; (**b**) Induced relative wavelength shifts of HE_1,m_ cladding mode when subjected to 500 g tension, plotted against unstrained wavelength at 475 °C.

For temperatures below 1300 °C, silica fiber-based high order Type II FBGs can be used for multi-parameter sensing. As previously shown in Equation (3), the non-sinusoidal profile of the grating ∆*n* produces higher order Bragg resonances [46] that will propagate differently for a given waveguide. If the example of a low wavelength cut-off fiber is taken, the *Λ_G_* needed for a fundamental *λ_B_* at 1550 nm is 535.5 nm. The inscription of a fs-IR Type II FBG with a pitch *Λ_G_* = 4.184 μm, that is 8 times larger than the pitch needed to produce a fundamental Bragg resonance in the fiber, creates 6 higher order resonances that can be observed in the wavelength window of 1 to 1.8 μm where the fiber is single mode [96] (see Figure 15a). According to Equation (3), the order numbers *m* of these resonances correspond to values from 7 to 12. Changes to *n_eff_* and hence *λ_B_* due to changes in the thermo-optic and strain optic coefficients are different for different wavelengths for a given waveguide. By using a single grating structure in this way, the cross sensitivity of strain and temperature can be removed by monitoring the high order resonances.

Multiparameter fiber grating sensing can also be realized by exploiting the differences in propagation between guided core modes and cladding modes that are generated when a FBG is written with a slight tilt of the grating planes with respect to the fiber axis (10°) [97]. Such tilted FBGs produce strong cladding mode resonances that are not core-cladding guided but instead are cladding-air guided. The tilted FBG produces strong losses that are observed in its transmission spectrum but these losses are easily affected by the environment surrounding the fiber. The wavelength shifts of both the core mode and cladding mode resonances are affected in the same way by temperature, however strain affects the cladding modes differently as compared to the core mode. A single tilted FBG can then be used as a dual parameter axial strain/temperature sensor. The Type I tilted FBGs reported in [97] were fabricated using UV-laser based techniques and therefore would not be effective at temperatures above 300 °C. The fabrication of tilted FBGs using the fs-IR laser and the phase mask approach was used to produce a dual parameter strain and temperature sensor that was operable up to 800 °C [98]. Instead of forming a tilted grating structure, thermally stable Type II fs-IR FBGs with strong coupling to cladding mode resonances can also be created by localizing the index modulation to the region near the core/cladding interface of the fiber [99,100,101]. These structures can also be used as multiparameter temperature/strain sensors at high temperature. An example of a Type II cladding mode multi-parameter sensor is shown in Figure 15b.

For temperatures above 1300 °C, such silica fiber-based multi-parameter sensors are not possible. Instead, the sapphire based FBG is a more appropriate choice for multiparameter sensing. For temperatures above 1000 °C, significant levels of thermal blackbody radiation are produced that can overwhelm the multimode FBG reflection signal [102]. This observed reduction in signal-to-noise due to the black body radiation can be overcome by interrogating the sapphire FBG in single mode as described earlier. The resulting single mode response from the grating is significantly stronger, because of the improvement in the signal-to-noise ratio. The blackbody radiation signal that was previously a detriment is now a benefit. By using the known temperature dependent blackbody radiation spectrum in sapphire as a temperature reference [78], temperature dependent shifts in the strong single mode Bragg resonance of the sapphire FBG can be decoupled from strain induced wavelength shifts. In this fashion, a dual parameter temperature/strain sensor was demonstrated [103].

### 3.4. High Pressure

FBGs written in the telecom band in standard Ge-doped silica fibers have been observed to undergo negative wavelength shifts of −3 pm/MPa when exposed to hydrostatic pressure up to 70 MPa [104]. The FBG response to pressure was improved by adhering to the FBG a transduction element consisting of a carbon fiber laminated composite structure. Pressure sensitivity was improved by 3 orders of magnitude while measured at room temperature and pressures up to 70 MPa [105]. 

Asymmetric waveguide geometries have been devised where changes in hydrostatic pressure will result in polarization dependent changes in the strain-optic coefficient. Exploiting these waveguide designs, it is possible to fabricate an FBG-based dual parameter fiber optic temperature/pressure sensor. For example, a UV-laser induced FBG was written into a side-hole optical fiber that was then used to measure simultaneously temperature and high pressure [106]. The presence of side air holes in the fiber cladding caused the fiber to be highly birefringent producing two polarization dependent *λ_B_*’s from the one FBG. As pressure increased within the side holes, changes to the wavelength separation of the polarization dependent *λ_B_*’s was observed. Temperature changes however caused identical wavelength shifts of both resonances. An 8.4 pm spreading of the Bragg resonances was observed for every MPa of pressure applied to the FBG. As the grating used was a Type I UV grating, it could not be used as a multi-parameter pressure/temperature sensor at temperatures greater than 300 °C.

With a similar side-hole waveguide geometry, a multi parameter pressure and temperature sensor was created using fs-IR Type II FBGs [107]. The resulting sensor functioned well at temperatures up to 800 °C and pressures up to 16.5 MPa. This approach relies on the waveguide-induced birefringence of the grating response rather than the structural birefringence associated with the nanograting structures of the Type II index change. Recently, a complex ‘butterfly’ microstructured photonic crystal fiber which possessed a roughly 3-fold increase in birefringence as compared to side hole fibers, was used to create a fs-UV Type I FBG combined pressure/temperature sensor for extreme pressure monitoring (up to 140 MPa and 290 °C) [108]. The increased birefringence of the novel photonic crystal fiber created a more sensitive pressure induced wavelength spread of the states of polarization of the Bragg resonance.

### 3.5. Through-the-Coating (TTC) FBG Inscription for High-Strain Measurements

The capability to measure high strain levels with optical fiber is desirable for structural health monitoring of high energy and aerospace systems. FBG based sensors typically cannot reach the high strain fields of up to ~50,000 microstrains (με) required for many of these applications because of the nature of typical UV-laser based FBG inscription methods. Most optical fibers have UV-absorbing protective polymer coatings which need to be removed before a FBG can be written. The coating removal and its reapplication aside from being labor intensive and time consuming processes, significantly weaken the mechanical strength of the fiber unless special precautions are taken. It is preferable if the strip and recoat processes can be avoided. Special UV-transmissive fiber coatings have been demonstrated in order to perform through-the-coating (TTC) inscription of UV-laser based FBGs [109]. 

Inscription of gratings with single pulses from high-power lasers directly on the draw tower before application of the protective polymer [110] is an effective technique for fabrication of FBG sensor arrays that can withstand high levels of strain [111,112]. Draw tower gratings (DTGs) are typically written using a nanosecond pulse duration UV laser source such as a line-narrowed excimer laser system which has a long spatial coherence to permit the usage of bulk interferometric exposure such as with a Talbot interferometer [113]. The limitation of DTGs is that because of the single-pulse nature of the exposure they tend to have low reflectivites (<10%) unless specialty UV photosensitive fibers are used. The critical draw tower infrastructure is also required. A TTC inscription method based on infrared femtosecond laser exposure is more flexible as it is not limited to UV photosensitive fiber types, most polymer coatings are transparent to IR light and much higher grating reflectivities are possible, thereby facilitating the use of ‘off-the-shelf’ fibers.

TTC inscription of FBGS using fs-IR laser radiation was first demonstrated using the PbP method [94] which produced thermally stable microvoid based FBGs. The high-NA microscope objective used in the PbP method easily created the large differential in exposure intensities that were needed to induce the grating in the fiber core but at the same time leave the IR-transmissive polymer coating unmodified. However, the PbP index change mechanism of single pulse microvoid formation significantly reduced the mechanical resilience of the fiber resulting in breakage levels that were roughly 1/3 of those of pristine fibers [114]. 

Femtosecond laser inscribed TTC FBGs can also be fabricated using the phase mask approach. Direct writing of Type I FBGs through the acrylate coatings of hydrogen-loaded SMF-28 fibers and the acrylate and polyimide coatings of high Ge-doped bend insensitive fibers were reported using a fs-IR laser and the phase mask technique [115,116,117]. The implementation of TTC FBG inscription using the phase mask method as compared to the PbP method relies on the usage of longer focal length lenses as opposed to a microscope objective. This lens geometry is needed in order to relax alignment constraints and accommodate positioning of the phase mask, focusing lens and target fiber. Cylindrical lenses with shorter focal lengths have shorter Rayleigh ranges thus greater intensity differences between the fiber core and the surface of the coating can be created. More strongly converging lenses however also have increased geometrical aberrations which produce larger than expected grating structures. This is a problem if index change needs to be localized in the core and at the same time be as far away from the surface as possible. In order to create a highly localized beam in the fiber core but at the same time have a large intensity differential between the core and protective coating covering the cladding, a lens free of geometric aberrations is needed. Using an acylindrical focusing lens which reduced geometrical aberrations and a phase mask, Type I TTC fs-IR FBGs were successfully written through acrylate and polyimide coating of Ge-doped and PSC optical fibers [118]. The generated Type I TTC FBGs possessed mechanical strength similar to that of the pristine fiber and could measure strains up to 60,000 με. 

Empirically, TTC writing of Type I fs- IR FBGs is improved by: (i) using tight focusing optics that create a large differential in intensity between the fiber coating and the fiber core, such as a multiple lens system [119] or an adapted acylindrical lens [118] thus reducing the required pulse energy; (ii) using a phase mask that produces a fundamental Bragg resonance in the fiber [115] but at the same time has good zero order suppression [120], and (iii) reducing the pulse duration of the femtosecond source (from 120 to 35 fs) [118].

**Figure 16:**
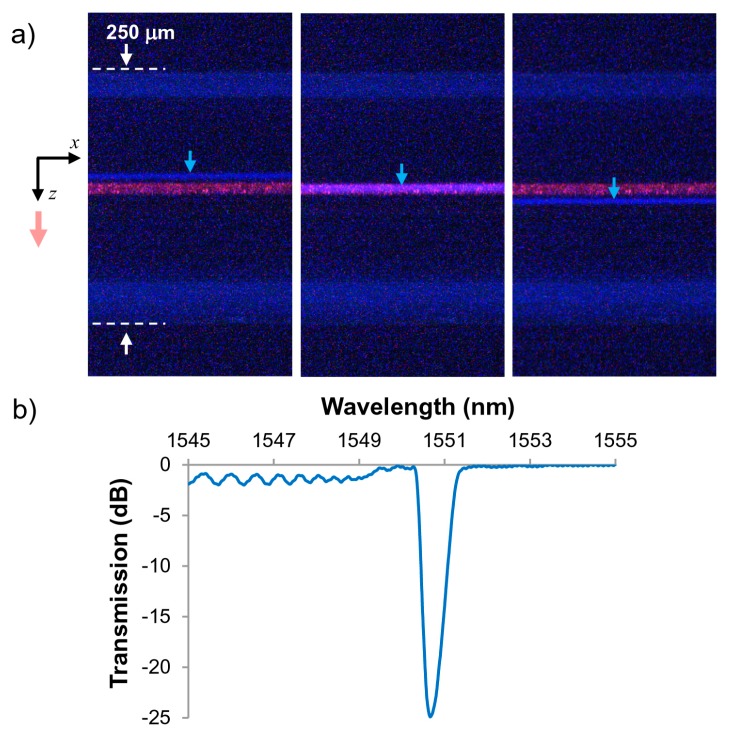
(**a**) Femtosecond laser focus alignment through an acrylate-coated *SMF-28* fiber as facilitated by nonlinear and dark-field microscopy [121]. The fiber core is imaged by Rayleigh scattering of coupled red *λ*_2_ = 637 nm light. The left-hand image shows blue photoluminescence in front of the core, the middle image the increased photoluminescence as the line focus overlaps the core, and the right-hand image the photoluminescence of the laser line focus beyond the core. (**b**) The transmission spectrum of a TTC FBG written with the photoluminescence line focus aligned on the core (middle image Figure 16a).

The employment of tight focusing optics necessitates the usage of an accurate and precise alignment method to ensure beam focusing at the fiber core and not on the fiber coating. Recently, we disclosed a nonlinear photoluminescence imaging technique that was used to visualize the intensity distribution of infrared femtosecond laser pulses inside the optical fiber during Bragg grating inscription using side illumination through a phase mask [121]. This technique, which results in direct imaging of the inscription laser field that induces nonlinear photoluminescence inside the optical fiber, facilitates (i) the characterization of the laser focus in the vicinity of the fiber core and (ii) the optimization of the fiber alignment with respect to the laser focus while using pulses with energies several times lower than those used during the actual inscription process. Figure 16 presents an example of how this technique is used to align the femtosecond laser line focus within the core of an acrylate-coated SMF-28 fiber. Using a 1.07 μm pitched phase mask that is optimized for 800 nm radiation but is specialty coated to suppress the transmission of the zero order and a 12 mm focal length acylindrical lens, the line focus of the interfered ±1 orders from the mask generates nonlinear photoluminescence in the fiber core and cladding. Pulse energies roughly half of those needed to induce a measurable grating were used. The core of the fiber is illuminated by launching red light which couples out of the core via Rayleigh scattering. Depending on the location of the femtosecond laser line focus within the fiber, the wavelength and intensity of the photoluminescence would vary [121]. In the silica cladding, for example, blue photoluminescence at ~460 nm was observed that is associated with non-bridging oxygen hole centers and oxygen-deficiency defects [122]. When the beam is aligned with the Ge-doped core however, much stronger 400 nm photoluminescence from the GeO defect is observed [123]. By maximizing this luminescence through positioning of the line focus as well as by adjusting mask, lens and fiber tilt, the optimal alignment for FBG inscription is achieved. 

This approach is quite versatile and allows one to exploit beam vignetting or transverse walk off of phase mask orders to inscribe complex grating structures such as ultrashort (~100 μm in length) TTC gratings in polyimide coated fiber for highly localized strain or acoustic measurements [124]. By varying the pulse energy and chirp, TTC Type II FBGs are easily fabricated [116,120] with index modulations up to 2 × 10^−3^, scattering losses below 0.1 dB and no visible damage to the polymer coating. For some applications, for example in the oil and gas sector, it is desirable to be able to inscribe a TTC grating that is thermally stable up to temperatures at least of the those rated for the protective polymer coating. In the case of polyimide, that is approaching 400 °C.

### 3.6. FBGs for Shockwave Detection in Energetic Materials

A chirped fiber Bragg grating (CFBG) has a varying periodicity of the grating pitch along the fiber length. Different locations along the grating will thus reflect different wavelengths of light. This grating attribute was used effectively in the telecommunication industry to correct for chromatic dispersion of optical signals that occurred in long-haul optical fiber networks. From a sensing perspective, such structures allow for truly continuous high spatial resolution measurements of, for example, strain and crack formation in a composite laminate [125], or temperature and strain distributions within photonic lightwave circuit packages [126]. For harsh environments, CFBGs when coupled to high speed interrogation systems are a robust approach for continuous spatial measurements of shock wave and detonation front propagation within energetic materials or explosives [127,128]. The propagation distance over which the shock wave can be measured is limited by the length of the CFBG. The length of commercially available CFBGs is limited by the length of the chirped phase mask that is used to inscribe them. Phase masks are usually manufactured through standard photolithographic processes and are therefore limited to ~150 mm in length. 

Femtosecond laser inscribed FBGs using the PbP method are not constrained by these limitations of the phase mask. In fact, it is straightforward to manufacture a chirped grating structure using the PbP method by simply varying in a linear way the pulse repetition rate of the inscription laser while the beam travels along the fiber at a constant velocity [51]. The issue, however, is that the microvoid formation process induces high scattering losses of ~1–2 dB/cm [129]. For a 100 mm grating, this would result in a total loss >20 dB with the device operating in the reflection mode, which is the case for shock wave velocity measurements. Recently, a technique to scan the focal spot along one axis of the fiber core produced a PbP styled grating that had low scattering losses of 0.05 dB/cm [44]. The pulse energies used were below that of the threshold for nanograting or microvoid formation (Type I regime), with scanning speeds and core dithering optimized to ensure some overlap between irradiating pulses. Using this technique, a chirped fiber grating with an average reflectivity of 40% across a 10 nm bandwidth and an overall length of 19.5 cm was created [130]. 

**Figure 17:**
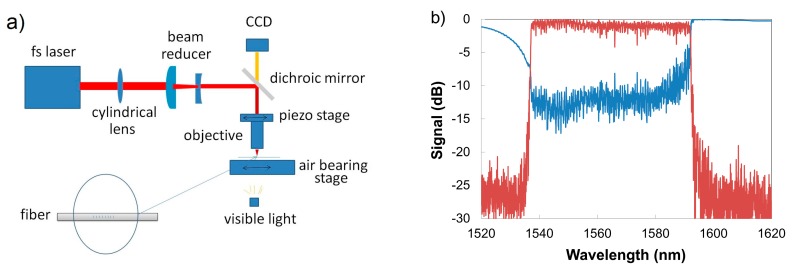
(**a**) schematic representation of the plane-by-plane exposure set up; (**b**) Transmission (blue) and reflection spectra (red) of the 26 cm long chirped fiber grating in SMF-28 fiber made using the plane-by-plane method.

In an alternative approach, the strongly asymmetric ‘tear drop’ shaped zone of refractive index change created by the microscope objective in the PbP process is modified by introducing a long focal length cylindrical lens in the beam path that has a co-incident focal position with that of the microscope objective (see Figure 17). The axis of the cylindrical lens is oriented perpendicular to the fiber axis such that beam waist is modified from a cylindrical shape to a planar strip or plane [131]. A variation of this astigmatic beam shaping approach using a cylindrical telescope is also reported [132]. Each pulse from the laser then produces a grating plane. The Type I chirped gratings were created using 800 nm 120 fs pulses from a regeneratively amplified Ti:sapphire laser having energies of 1.6 μJ. A beam reduction telescope consisting of one plano-convex lens and one plano-concave lens was placed after the cylindrical lens to reduce the laser beam diameter to match the pupil diameter of the microscope objective (50X/0.6, Nikon Instruments, Melville, NY, USA). The fiber (SMF-28) was mounted on an air bearing stage (Aerotech) that moves with a positional accuracy of ±10 nm. The laser repetition rate was set at 250 Hz and the fiber was translated at a speed of 132 µm/s resulting in a fundamental 1st order grating period of 0.528 µm. The grating chirp was created by varying slightly the laser repetition rate from 250 Hz to 248.7 Hz in 0.02 Hz steps while the fiber sample was translated at a constant velocity. In this fashion, a 26 cm long chirped FBG was fabricated in standard telecom fiber (i.e., SMF-28), which had a chirp rate of 2 nm/cm, a 3 dB bandwidth of 55 nm, reflectivity of >60% across the band and an overall scattering loss of less than 0.5 dB. The plane of type I index change that subtends the SMF-fiber core was measured with an optical microscope to be approximately 11 μm in diameter but less than 300 nm thick. Transmission and reflection spectra are presented in Figure 17b. The reflection spectra shown in Figure 17b are not smooth. This is likely due to the phase errors generated in the grating by the air-bearing stage with its position error of ±10 nm [133]. A flatter reflection spectrum would be achieved if the grating strength is improved. This can be achieved by having a fiber core with a larger Ge content or by loading the fiber with H_2_ or D_2_ [32].

### 3.7. Sensing with fs-FBG-Based Fiber Lasers

The sensing applications presented above rely on the grating structure being interrogated remotely in a passive way. Fs-IR FBGs can be easily inscribed in active rare-earth doped fibers in order to make active sensor devices based on fiber laser designs. Fs-IR FBGs that are intrinsic to an active fiber can act as resonator cavity mirrors in a fiber laser either in a distributed Bragg reflector (DBR) or distributed feedback (DFB) configuration. In the DBR case, the fiber laser cavity is comprised of two FBGs with a given physical separation and overlapping *λ_B_*’s. In the latter DFB case, the introduction of a π-phase shift in the grating structure causes the single grating to act as fiber laser resonator cavity. The resulting laser emissions from these fiber laser configurations have much narrower spectral bandwidths than the Bragg gratings. FBG-based fiber lasers have been used as acoustic sensors or more specifically hydrophone and magnetic field sensors because of their improved spectral resolution and high signal to noise ratio. When interferometically interrogated, these fiber laser based sensors have achieved strain resolutions approaching 10’s of fε/√Hz [134]. Using the DBR configuration, FBG cavity mirrors can be separated by meters causing the strain measurement to be integrated over the length of the laser cavity. These fiber laser based active sensors have only been realized with Type I FBGs. For high temperature or high optical power conditions Type I FBGs will not withstand the harsh conditions. Thermally stable regenerated gratings were used to create a fiber laser cavity that could operate at high temperature [135]. Regenerated gratings typically have low reflectivity and therefore are not appropriate for some fiber laser cavity configurations. 

**Figure 18:**
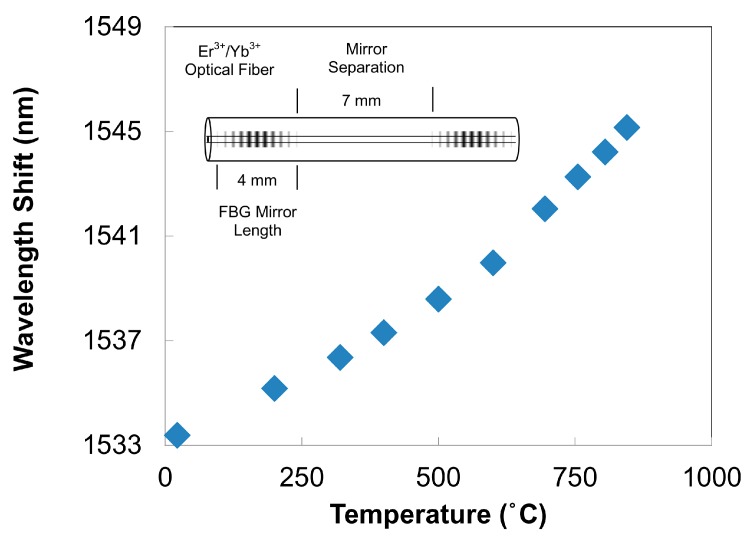
Wavelength variation of a fiber laser cavity comprising fs-FBGs with temperature. Inset: schematic of the fiber laser cavity.

The Type II fs-IR FBGs written in actively doped silica fibers are excellent candidates for intrinsic cavity mirrors that can operate at high temperatures ranging from 600 to 1000 °C. FBGs written using the PbP technique were used to create a laser cavity 3 cm in length that was operable up to 600 °C [136]. Using the phase mask approach, very short laser cavities (~7 mm) were made in heavily doped active Er-Yb fiber (475 dB/m loss at 975 nm and 26 dB/m loss at 1535 nm) using high reflectivity fs-IR Type II FBGs [137]. The laser cavities had a 10% lasing efficiency when pumped with a 500 mW 980 nm laser diode and were tested up to 850 °C. The free spectral range of the laser cavity is ~200 pm which is narrower than the 670 pm bandwidth of the cavity mirrors. This resulted in occasional mode hoping. As shown in Figure 18, the laser emission wavelength shifted as a function of temperature. It is likely that non-uniformities in heating along the fiber cavity length caused the cavity mirror reflection wavelengths to walk off of each other above 850 °C. A chirped high reflector cavity mirror or a DFB configuration may be more favorable to avoid the adverse effect of the temperature gradient.

## 4. Conclusions

In this review paper, recent developments in manufacturing of femtosecond laser induced fiber Bragg gratings and their application for harsh environment sensing are presented along with some of the background theory of femtosecond laser-material interaction physics. Bragg grating written into silica based optical fibers with femtosecond infrared and visible lasers using either the phase mask method or the point-by-point method can be used for sensing in harsh environment at temperatures less than 1000 °C if appropriate exposure conditions are used. Employment of exotic grating or fiber geometries can allow for the sensing of different parameters for a single FBG element. Gratings written in glass fibers such as pure silica core, radiation hardened fluoride-doped silica or pure silica microstrutured photonic crystal fiber can be used for sensors in the oil and gas, or nuclear industries where losses in standard optical fiber due to hydrogen ingress or ionizing radiation can significantly reduce sensor lifetime. Above 1300 °C extreme temperature measurement with Bragg gratings is relegated to sapphire optical fiber. In the case of sapphire FBGs, these robust devices are suitable for harsh combustion environments such as jet engines, coal gasification reactors, and natural gas turbines for electrical power generation. Finally, through-the-coating fiber gratings can be made with infrared femtosecond lasers to withstand high mechanical strains similar to that of the pristine fiber making them useful for high mechanical stress applications such as structural health monitoring of aircraft.
